# Feature Extraction and Classification Methods for Hybrid fNIRS-EEG Brain-Computer Interfaces

**DOI:** 10.3389/fnhum.2018.00246

**Published:** 2018-06-28

**Authors:** Keum-Shik Hong, M. Jawad Khan, Melissa J. Hong

**Affiliations:** ^1^Department of Cogno-Mechatronics Engineering, Pusan National University, Busan, South Korea; ^2^School of Mechanical Engineering, Pusan National University, Busan, South Korea; ^3^Early Learning, FIRST 5 Santa Clara County, San Jose, CA, United States

**Keywords:** brain-computer interface, electroencephalography, functional near-infrared spectroscopy, locked-in syndrome patient, feature extraction, classification

## Abstract

In this study, a brain-computer interface (BCI) framework for hybrid functional near-infrared spectroscopy (fNIRS) and electroencephalography (EEG) for locked-in syndrome (LIS) patients is investigated. Brain tasks, channel selection methods, and feature extraction and classification algorithms available in the literature are reviewed. First, we categorize various types of patients with cognitive and motor impairments to assess the suitability of BCI for each of them. The prefrontal cortex is identified as a suitable brain region for imaging. Second, the brain activity that contributes to the generation of hemodynamic signals is reviewed. Mental arithmetic and word formation tasks are found to be suitable for use with LIS patients. Third, since a specific targeted brain region is needed for BCI, methods for determining the region of interest are reviewed. The combination of a bundled-optode configuration and threshold-integrated vector phase analysis turns out to be a promising solution. Fourth, the usable fNIRS features and EEG features are reviewed. For hybrid BCI, a combination of the signal peak and mean fNIRS signals and the highest band powers of EEG signals is promising. For classification, linear discriminant analysis has been most widely used. However, further research on vector phase analysis as a classifier for multiple commands is desirable. Overall, proper brain region identification and proper selection of features will improve classification accuracy. In conclusion, five future research issues are identified, and a new BCI scheme, including brain therapy for LIS patients and using the framework of hybrid fNIRS-EEG BCI, is provided.

## Introduction

The primary function of a brain-computer interface (BCI) is to provide a means of communication for patients with the real world. A large proportion of the patients targeted for BCI applications are those who cannot control their muscle movements voluntarily, particularly the patients defined as “locked-in.” However, most current BCI research involves movement controlling devices with brain signals from healthy subjects. Patients with movement disorders may not be able to generate commands as effectively as healthy subjects, hence they may not be able to control these devices with high accuracy (McFarland and Wolpaw, 2010, 2011; Pan et al., 2014; Visani et al., 2015). Locked-in patients can use only cognitive functions, such as mental counting, listening, and imagination. In this paper, a new framework for hybrid feature extraction and classification for patients in the locked-in state is proposed by which they may be able to achieve smooth control of external devices such as a robotic arm or wheelchair in a real-world environment (Muller-Putz et al., 2015; Naseer and Hong, 2015a).

To find a solution for this problem, we first need to understand the components of a BCI (Banville and Falk, 2016; Ramadan and Vasilakos, 2017) that can be modified to achieve better solutions. The brain signals for a BCI are usually acquired either directly from the electrical activity of the brain or from the secondary route known as hemodynamics. Electroencephalography (EEG) signals reflect electrical activity originating as a result of neuronal firing when a task or activity is performed (Olejniczak, 2006). This activity is measured as differences in voltage between different locations on the surface of the head. The differences are caused by postsynaptic potentials in the cell membranes of cortical neurons. On the other hand, hemodynamic activity appears in the form of blood flow changes that result from neuronal firing, which can be measured by functional near-infrared spectroscopy (fNIRS) (Matthews et al., 2008; Min et al., 2010). Blood flow increases in an area of activated neurons at a greater rate than in areas of inactive neurons. The increased blood flow results in a surplus of oxyhemoglobin in the veins of the active area and a distinguishable change in the local ratio of oxyhemoglobin to deoxyhemoglobin. In the current literature, EEG and fNIRS are the only two modalities used for non-invasive BCI for locked-in patients (Hong and Khan, 2017).

As a modality for BCI, EEG is the most common method of recording neuronal signals, due to its portability. The signals most frequently used for BCI derive from motor imagery (MI), steady-state visual evoked potentials (SSVEP), and the P300 evoked potential (Trejo et al., 2006; Turnip et al., 2011; Turnip and Hong, 2012; Wang et al., 2012, 2016; Gruzelier, 2014; Ahn and Jun, 2015). These three signals have been used for controlling wheelchairs (Wang et al., 2014; Ramli et al., 2015; Zhang et al., 2016) and quadcopters (Kim et al., 2014). In addition to EEG, fNIRS is another widely used modality for BCI. Recent studies have shown the portability of fNIRS, thus making it a viable option (Santosa et al., 2013; Bhutta et al., 2014; Boas et al., 2014; Hong et al., 2015). Even though no study has yet shown the ability to control a wheelchair using fNIRS alone, several projects have used fNIRS to generate multiple commands (Power et al., 2012a; Naseer and Hong, 2013; Naseer et al., 2014). In some studies, EEG and fNIRS have been combined to improve classification accuracy (Fazli et al., 2012; Safaie et al., 2013; Tomita et al., 2014; Buccino et al., 2016). For locked-in patients, several projects have used hybrid systems of this type to decode brain activity for control and brain imaging (Blokland et al., 2014; Dutta et al., 2015; Das et al., 2016). However, there is still a large gap between the currently achievable performance and the accuracy needed for a practically usable interface for locked-in patients. Taking into account both non-invasiveness and portability, currently fNIRS and EEG form the best available combination of modalities for BCI. Methods of command generation methods using these modalities are the focus of this review.

The first issue to consider is selection methods for monitoring the best brain regions of interest. Most BCI studies use the international 10–20 (or 10–10) system for placement of EEG electrodes (Homan et al., 1987; Jurcak et al., 2007). Because EEG responses such as the P300 are highly variable across brain areas, channel selection algorithms are applied to determine a region of interest (ROI) (Feess et al., 2013; Alotaiby et al., 2015). In fNIRS, channel averaging is used because the hemodynamic responses are not distinctive among channels (Bhutta et al., 2015; Naseer and Hong, 2015b; Liu and Hong, 2017). These methods may be effective for able-bodied persons, but not necessarily for locked-in patients, since their brain activity patterns may not be correctly identified. Smith and Delargy (2005) found that the cerebral cortex of a locked-in patient might not show clear evidence of activation. Therefore, it is desirable to improve brain region selection methods.

Next, selection of the best type of brain activity to use for generating control commands can be an issue. A healthy person can perform a wide variety of tasks easily. Thus, an able-bodied person usually has a better understanding of the experimental paradigm and the activities that are to be performed. Many EEG-based wheelchair control examples are available in the literature, but of 35 recent studies on wheelchair control using EEG (Fernández-Rodríguez et al., 2016), no study investigated a locked-in patient. As most of these studies employed SSVEP- and P300-based schemes, the participants could adapt themselves to the scheme quickly (Hwang et al., 2012, 2013; Li et al., 2013a; Fan et al., 2015). However, for a locked-in patient, it is difficult to concentrate on a screen to receive stimuli and generate commands. Therefore, a second aspect to consider is the type of BCI: active, reactive, or passive. The majority of BCIs have been designed using reactive tasks (Zander and Kothe, 2011). In these cases, the stimuli are given in the form of an audio, video or pain cue (Hu et al., 2012; Zhang et al., 2013, 2014; Hong and Nguyen, 2014; Santosa et al., 2014). These result in high accuracies when used with normal, healthy subjects. Again, however, it can be difficult for a patient to concentrate on such stimuli. An active-type BCI may be more effective for these patients. For example, a Yes/No decoding scheme has been successfully implemented for patients using a non-invasive active BCI (Chaudhary et al., 2017). Another point to consider is the number of distinct commands the patients can perform.

The third issue is how to convert the detected brain signals into machine commands. Identifiable features contained in brain signals need to be defined, extracted, and classified to translate the analog brain signals into digital commands (Ortiz-Rosario and Adeli, 2013). Because different types of brain activity patterns can be decoded from locked-in patients, the feature selection and classification criteria developed for healthy people may need to be reevaluated. It is not clear yet whether the same feature sets used for healthy persons will work for patients (Naseer et al., 2016a). However, if proper features are selected, conventional classifiers (e.g., Bayesian, linear discriminant analysis) may complete the job. Moreover, to achieve fault-tolerance, it would be better to use both neuronal and hemodynamic features simultaneously (Hong and Khan, 2017).

**Figure 1:**
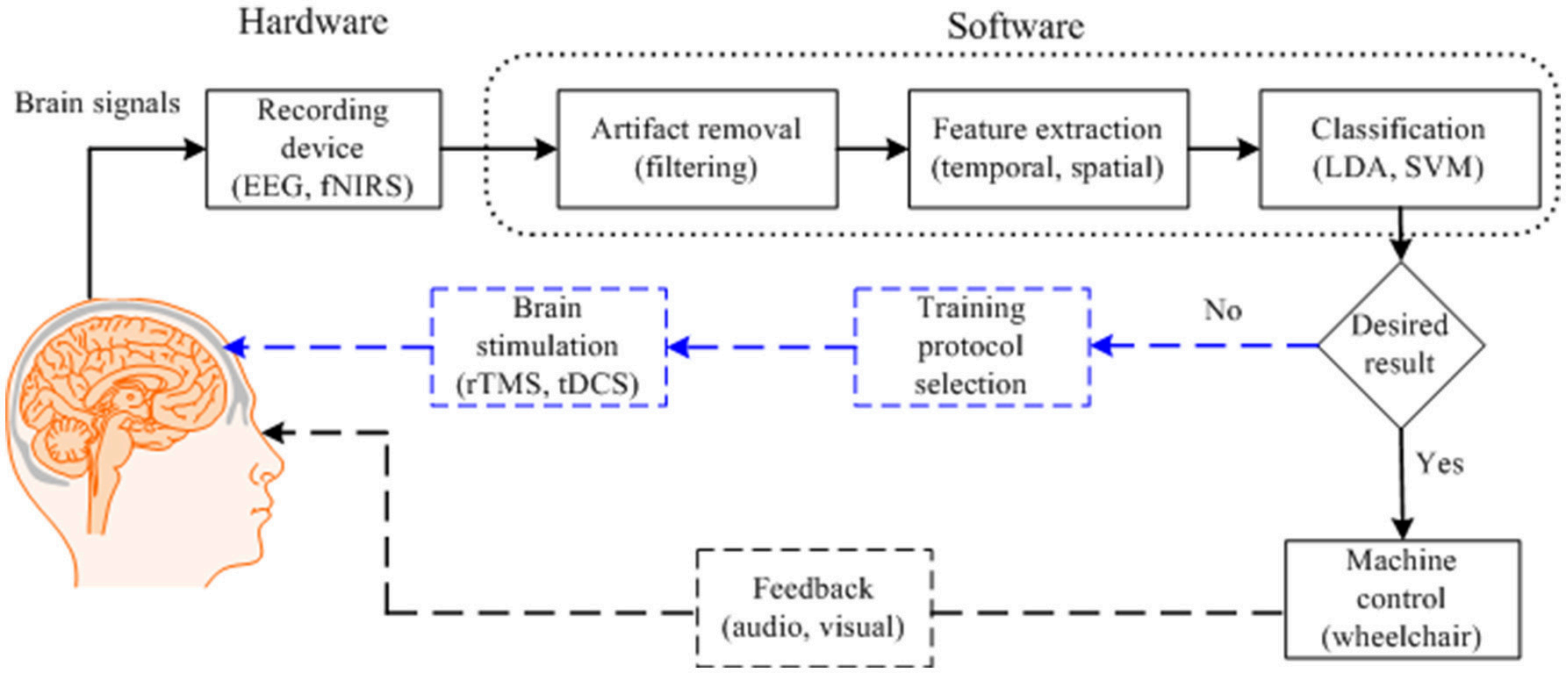
Typical brain-computer interface scheme for control applications with brain function recovery.

Figure 1 depicts a typical BCI scheme, including signal acquisition, filtering, feature extraction, classification, and interfacing to external devices. After classification, a control interface completes the system. An average classification accuracy of at least 70% is essential for practical use. Most experiments conducted in a laboratory environment using able-bodied subjects could achieve accuracies higher than this. However, the accuracy drops drastically in the case of patients. If the necessary accuracy is not achieved in the early stages, physical stimulation of the brain may be useful. To improve the results for patients (e.g., stroke patients), additional measures using repetitive transcranial magnetic stimulation (rTMS) or transcranial direct current stimulation (tDCS) to improve brain functions may be needed (Dutta, 2015; Dutta et al., 2015). In general, the classification accuracy and the number of distinguishable commands can be improved by i) combining signals from a non-brain signal acquisition modality (e.g., a camera) with those from a brain signal acquisition modality, ii) developing a predictive model for early detection of a brain signal from a slow modality (e.g., fNIRS) in combination with a fast modality (e.g., EEG), and iii) identifying multiple cognitive activities simultaneously by integrating neuronal and hemodynamic signals.

In this review, we discuss the feasibility of hemodynamic and hybrid methods that can provide better results for achieving a high accuracy for BCI. In section What Patients Should BCI Focus?, the types of locked-in patients are categorized to clarify the brain regions that can most usefully be targeted in locked-in patients. In section Brain Activity Selection, channel selection schemes for brain region identification that can be used for these patients are reviewed. In section Methods to Determine the Region of Interest, the types of brain activities that can be used by locked-in patients for control are discussed. In section Feature Extraction and Classification Criteria, features that may provide more compatibility for command generation are examined. Moreover, classification techniques are examined. Section Device Interfaces discusses external device interface techniques. Finally, in section What Future Direction to Adopt?, our own scheme for BCI (for, e.g., multiple choice selection or wheelchair control) is proposed, and the challenges involved in implementation of the scheme are discussed. In this review, the primary foci are fNIRS-based BCI and hybrid fNIRS-EEG-based BCI, because several reviews of EEG-based BCI, including brain region selection and feature extraction, are available in the literature. However, EEG-based schemes are briefly explained where necessary.

## What patients should BCI focus?

Patients can be categorized based on their conscious state, attentional state, executive functions, intellectual ability, perception, and visual and verbal memories. However, the two aspects most commonly used to categorize patients are motor state and cognitive state (Guger et al, 2017). In general, BCI is well suited for patients who have limited motor activity but good cognitive skills. BCI may not be as effective for those in impaired cognitive states, as they may not be able to understand or perform the mental tasks used for controlling a device. Table 1 shows a classification of patients based on motor and cognitive states. By examining the table, we can see that BCI is probably suitable for patients with locked-in syndrome (LIS) and completely LIS (CLIS). Studies have shown that patients with LIS sometimes progress to the CLIS state. BCI research should target these patients. According to Nicolas-Alonso and Gomez-Gil (2012), a high grade of disability among LIS patients forces them to use BCI rather than relying on conventional interfaces. In any case, the first step is to determine the type of patients for whom a BCI is essentially needed.

**Table 1:** Categories of patients based on motor and cognitive states (Guger et al, 2017).

		**Cognitive state**			
		**No cognition**	**Minor cognition**	**Major cognition**	**Normal**
Motor state	No response	Comma patient		Completely locked-in syndrome patient (CLIS)	
	Minor motor response	Unresponsive wakeful state (UWS)	Minimal conscious disorder (MCD)	Locked-in syndrome patient (LIS)	
	Major motor response			Motor impairment patient (MI)	
	Normal		Cognitive impairment patient (CI)		

*BCI can be pursued for patients in the shaded area*.

The study of Kennedy and Adams (2003) categorized patients into six types. Four out of the six types showed detectable brain activity: Type (i) patients are capable of movement; type (ii) patients are incapable of movement but show some detectable motor activity due to partial muscle movements; type (iii) are locked-in patients with no muscular activity signals but with detectable eye-movement; and type (iv) are completely locked-in patients. The remaining two types are not suitable for non-invasive BCI. The fifth type is patients in whom implanted electrodes can detect brain signals (even though EEG electrodes cannot), and the sixth type includes those whose brain activity is non-detectable. Since our focus is on BCI using non-invasive methods, we will discuss only the first four types of patients.

### Patients capable of movements

These patients are probably not suitable for BCI, because at least some of their motor functions are still intact. The brain states of these patients are working properly (Kawase et al., 2017). For those who cannot move their lower limbs, they can generate commands by conventional methods (e.g., pressing a button/switch) using their fingers to control a wheelchair.

### Patients with minor muscular movements

These patients may not have visible motor movements. However, some muscular movements (e.g., minor shoulder flexion/extension) are detectable. In these patients, the motor cortex is working properly, and the minor muscular movements are caused by signals sent from the brain to the muscles (de Oliveira et al., 2017). Most commonly, such movements are detectable by electromyography (EMG), which can be used for control purposes. Thus, with an appropriate interface, these patients are capable of controlling devices with partial muscular movements. In this case, generation of a sufficient number of commands to handle the required number of degrees of freedom is essential (e.g., for control of a wheelchair). Hybridization (e.g., EEG or EMG in addition to EMG) can be used to increase the number of commands available.

### Patients with minor eye movements

These patients may not be able to control most of their motor functions, but can perform very minor but detectable eye movements. In this case, the motor cortex is partially working (Käthner et al., 2015). The level of eye movement is an important factor. If the eye movement is significant, electrooculography (EOG) can be used to generate commands based on eye-movements. However, it may be difficult for these patients to concentrate on eye movements over long periods of time. Moreover, control of a wheelchair using eye movements may not be an effective strategy, because it interferes with the ability of the subject to watch the environment. For these patients, the use of a hybrid scheme will be a better option. If multiple commands are needed, EOG can be combined with EEG to increase the number of commands. Furthermore, SSVEP- and P300-based stimuli can be used to generate multiple commands and to control external devices. It would be desirable to generate commands using reactive visual stimuli, and using EOG for improved control.

### Fully-locked in patients

These patients cannot perform any type of motor activity. Any patient below this condition is in the vegetative state (VS). In these case, the motor cortex in the brain is not working, but these patients can perform cognitive functions (Kübler et al., 2001, 2005). A BCI is the most effective solution for such patients. The prefrontal cortex may be the best option for generating commands. It may be difficult for these patients to control a wheelchair, but “Yes/No” decoding based on a choice selection can be performed. Moreover, to achieve better results, a hybrid EEG-fNIRS interface may be best, as it gives the richest signals.

**Figure 2:**
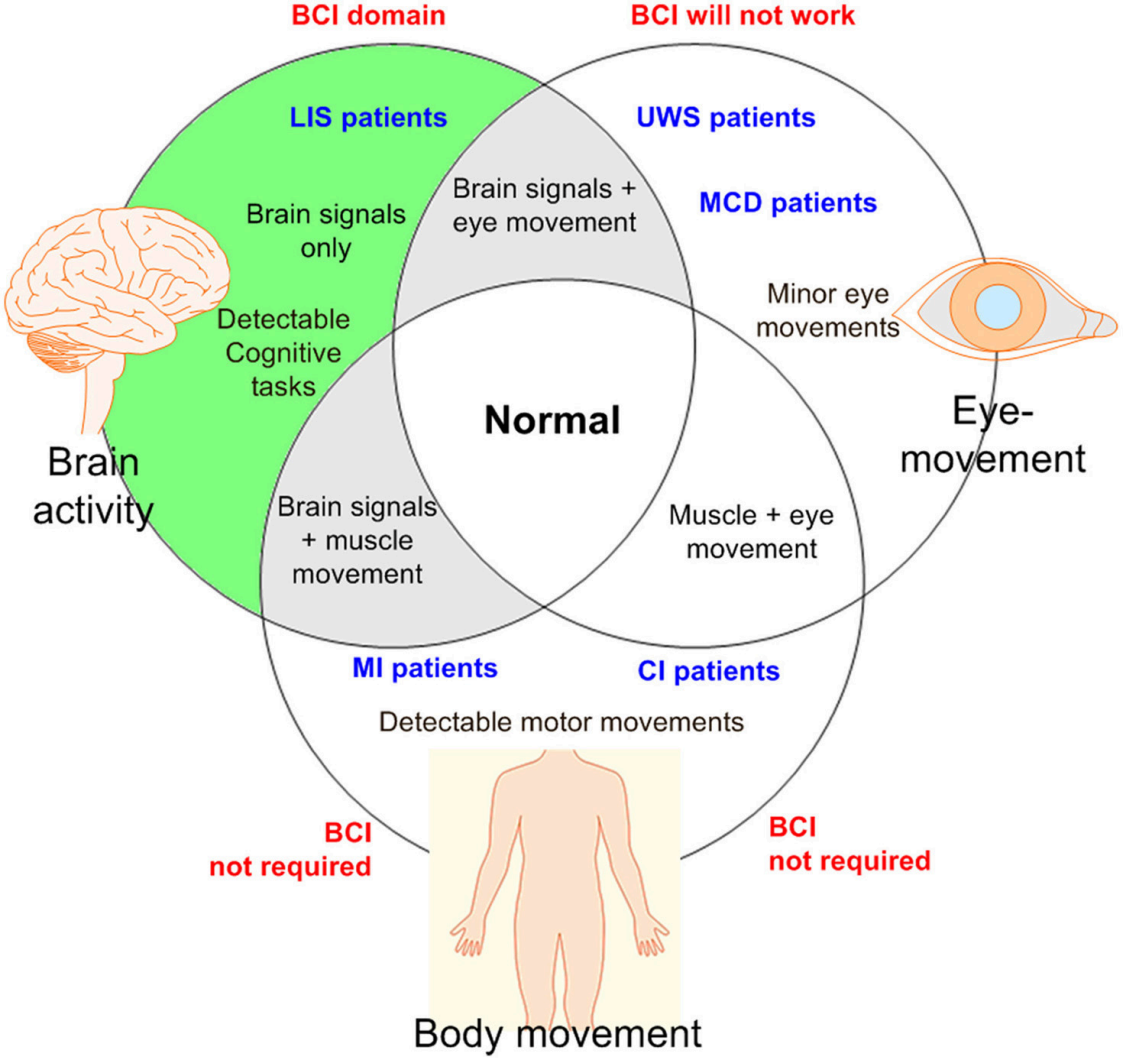
An illustration for BCI domain: BCI is required if there is no detectable muscular movement (BCI, brain-computer interface; LIS, locked-in syndrome; UWS, unresponsive wakeful state; MCD, minimal conscious disorder; MI, motor impairment; CI, cognitive impairment).

Figure 2 shows a categorization of patients based on the types of movements and the types of mental tasks that may be useful to generate commands for control applications. Based on the discussion above, it is emphasized again that a BCI is suitable for patients who have lost motor functions.

## Brain activity selection

The brain signals suitable for use with LIS patients have been discussed in several papers (Nicolas-Alonso and Gomez-Gil, 2012; Moghimi et al., 2013). However, only general mental and motor tasks were discussed, and the focus was on neuronal signals (EEG-based). As discussed in section What Patients Should BCI Focus?, signals generated from the prefrontal cortex may be the best choice for LIS patients. For partially locked-in patients, motor cortex activity can be considered as well, since the total number of cognitive functions that can be used for BCI purposes may be limited. The study by Weyand et al. (2015a) found that eleven different patterns of activity could be decoded from the prefrontal cortex. Therefore, focusing on the hemodynamic response, the following types of brain activity may be effective for partially and completely locked-in patients:

### Prefrontal activity

Prefrontal cortex signals are the cognitive activity patterns that are most suitable for LIS patients, as no motor task is involved. These signals may be generated by simple calculation or imagination tasks, and can be used in an fNIRS-based BCI. As discussed earlier, the study by Weyand et al. (2015a) provided the list of eleven activity patterns that can be detected from the prefrontal cortex. In a later study, a total of thirteen tasks are shown to be distinguishable in the prefrontal cortex using fNIRS (Naito et al., 2007; Naseer and Hong, 2015a). They are briefly discussed as follows.

#### Mental arithmetic

This task uses a simple mathematical calculation to generate the brain activity. Usually, a two-digit number is required to be subtracted from a three-digit number. This may be the most effective prefrontal active task; many studies have reported its efficacy for generating a command. One study reported that such brain activity could be made more prominent by increasing the complexity of the mathematical problem (Verner et al., 2013). The use of this task has been reported in both an fNIRS-based BCI (Bauernfeind et al., 2008; Power et al., 2012b) and a hybrid EEG-fNIRS-based BCI (Khan et al., 2014; Khan and Hong, 2017).

#### Mental counting

This is another mathematical task, similar to the mental arithmetic task above. Although both appear in the same region of the brain, mental counting task takes on a different form/feature in the prefrontal cortex. In the case of mental counting, a patient is asked to count numbers backward from a three-digit number for a given duration slowly while relaxing. In comparison to mental arithmetic, mental counting is an easier task for patients (Naseer and Hong, 2015b).

#### Mental singing

The mental singing task involves imagining oneself singing a song. Not many studies have used this type of task for research. As per the literature, the resulting activity appears in the prefrontal brain region (Power et al., 2010, 2011).

#### Word formation

The activity-generation method for this task has been described in three different ways. One approach is to give a specific letter to the subject (e.g., “d”) and ask them to form words (e.g., “door,” “deer,” “desk” using “d”) (Faress and Chau, 2013). The second approach is to give a scrambled 6–7 letter word (e.g., “tocekr”) and ask the subject to form the correct word (“rocket” in this example) (Khan and Hong, 2017). The third is to ask the subjects to recognize a letter displayed on a screen (Hofmann et al., 2008). The third method has shown promising results with patients.

#### Puzzle solving

For this, a screen is required to give a stimulus. A tangram puzzle is shown on the screen and the subjects are asked to imagine a puzzle solution by rotating the pieces. A few studies have reported significant results using this puzzle-solving paradigm (Zafar and Hong, 2017).

#### Mental rotation

Similar to mental counting, mental rotation is a type of puzzle-solving task. In this case, an object is shown on a screen, and the subjects are asked to imagine the rotation of the object (Abibullaev and An, 2012; Qureshi et al., 2017). To some patients, this might be an easy task to perform.

#### Happy thoughts

This may not be a very effective task, as only two studies have reported using it to drive prefrontal activity (Tai and Chau, 2009). The brain activity is detected when one imagines a past happy event. It may be a possible option for LIS patients, but further research is required.

#### Stroop test

For this case, the participants are shown a series of color names. The color names are each written in a single color, but the colors named by the words do not always match the colors they are written in. For example, the word “green” may be written in blue. The participants asked to think about the name of the color that the word is written in. This task has been used in a few fNIRS studies (Schroeter et al., 2002; Ehlis et al., 2005).

#### Future visualization

The subjects are asked to imagine their life after 5 years, specifically focusing on day-to-day activities. There is a lack of evidence on the suitability of this task for use with patients. Further research is required (Buckner et al., 2008).

#### Focus

Usually, in this type of task, the subject is asked to focus on a screen. Most commonly, the subjects are asked to scrutinize signals appearing on the screen, which could be brain signals or pictures of a ball moving on the screen. This may be not an effective task, as it is difficult for LIS patients to focus on a screen (Izzetoglu et al., 2011).

#### Motor imagery in the prefrontal cortex

Motor imagery is a motor function-related activity that can be defined as imagination of a movement of a part of one's own body, without any actual movement. According to the current literature, an activity related to motor imagery appears in the pre-motor cortex region (Holper and Wolf, 2011; Stangl et al., 2013; Kaiser et al., 2014). Some studies also indicate that it appears in the prefrontal cortex (Hatakenaka et al., 2007; Leff et al., 2011; Weyand et al., 2015a). Although a healthy person can perform motor imagery, it is often difficult for a disabled person to do so. Since the primary objective of a BCI is to form a communication pathway for motor-disabled people, it is a problem that only limited numbers of patients can perform this task. Both EEG and fNIRS are good options for detection motor imagery. For patients with tetraplegia, a hybrid EEG-fNIRS scheme is the best option for detection of motor imagery (Blokland et al., 2014).

#### Picture imagery

Like musical imagery, the brain activity is generated by imagining a picture. This task was used in an early fNIRS-based BCI (Naito et al., 2007).

#### Other

Several studies have reported that brain activity increases if subjects are asked to relax and put their minds to rest (Schudlo et al., 2013, Schudlo and Chau, 2014).

**Figure 3:**
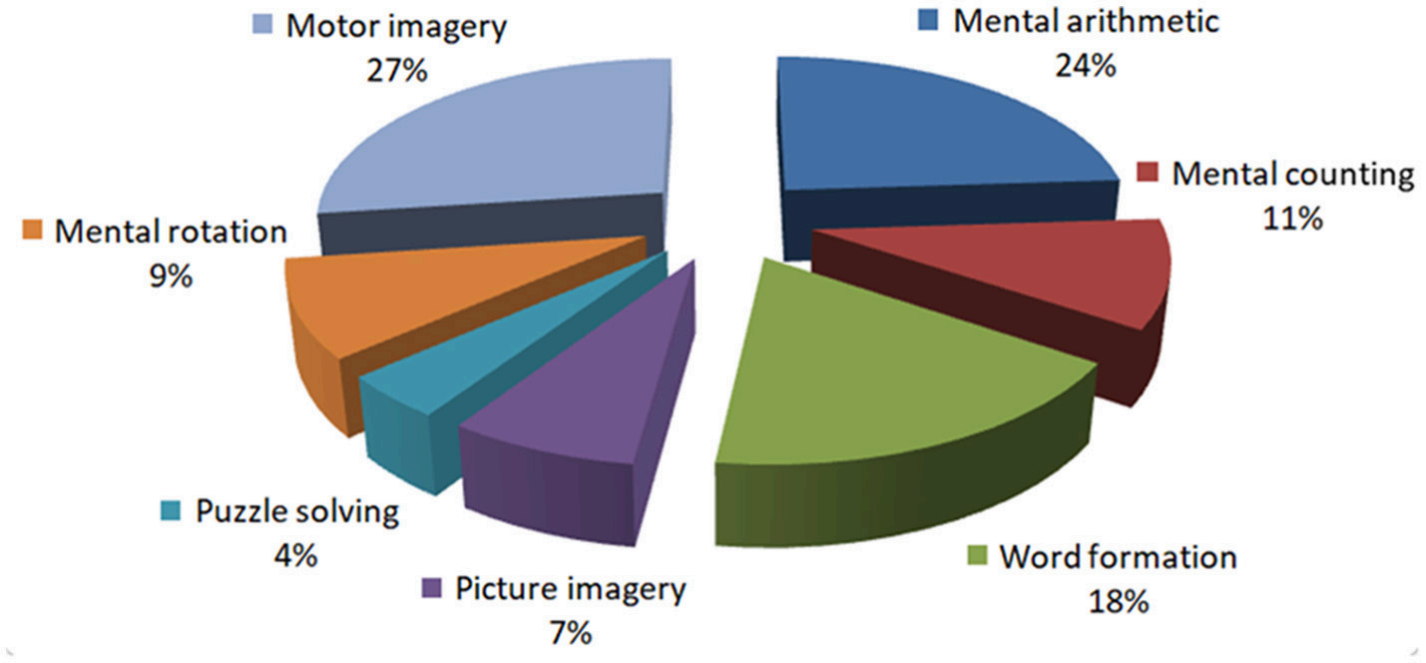
Distribution of the prefrontal tasks used for brain-computer interfaces: This chart was constructed using 102 papers (2002–2017) from the Web of Science (www.isiknowledge.com).

In the fNIRS-BCI literature, there are only two studies that have used patients to record the brain signals (see **Table 3**): Mental arithmetic and metal listening based tasks were used in these two studies to measure the hemodynamic signals. Since listening is a reactive brain activity that requires external stimuli to generate the brain activity, a mental arithmetic task (being an active task) can be a better option for a patient. However, the literature has no conclusive evidence about the best task for patients for BCI. The pie-chart in Figure 3 shows the distribution of tasks that have appeared in the literature (2002–2017, 102 articles). This may give an overall idea of the possibilities for the selection of an activity for patients to perform.

## Methods to determine the region of interest

It is obvious that if the wrong brain region is selected, proper signals for a BCI cannot be obtained. If we know the exact brain location to be monitored, the burden of placing heavy caps/electrodes/optodes for activity detection can be avoided. A healthy subject may be able to perform the given tasks correctly even using a complex brain signal acquisition system; however, a patient may not be able to do any sort of activity while wearing heavy head-gear. The research issue in this regard is how to select a small brain region, so that the BCI system can be controlled using a limited number of electrodes/optodes. A very comprehensive study of EEG electrode selection has been performed by Alotaiby et al. (2015). However, for fNIRS, no study has fully explored channel selection methodologies yet. Therefore, in this paper, strategies for fNIRS channel selection, which plays a vital role for LIS patients, will be the focus of attention.

### Algorithms for determining the region of interest

There are several algorithms that can be used to select the region of interest for BCI. The methods reported in the literature when choosing meaningful fNIRS channels are discussed below:

#### Channel averaging

This is the simplest approach, adopted in a number of fNIRS BCI studies (also in EEG and hybrid EEG-fNIRS studies) (Sagara and Kido, 2012; Naseer and Hong, 2013; Scarpa et al., 2013; Naseer et al., 2014). Here, all the channels used to detect brain activity are averaged. Equation (1) shows the channel averaging scheme:

(1) $$\begin{array}{l} \Delta HbX_{avg}\left(k\right)=\frac{\overset{M}{\underset{j=1}{\sum }}\Delta HbX\left(k,j\right)}{M}, \end{array}$$

where *HbX*∈ {*HbO, HbR*} represents a type of hemoglobin, *M* is the total number of channels, *k* represents the discrete time for which the signal is recorded, and *j* represents the channel number. This technique has a drawback in the case that the brain activity appears in only a few channels and the remaining channels do not show any activation. Averaging over the inactive channels significantly reduces the intensity (peak) of the data. This also leads to a significant drop in accuracy. Thus, this scheme may not be suited for LIS patients.

#### Averaging over a local region

**Figure 4:**
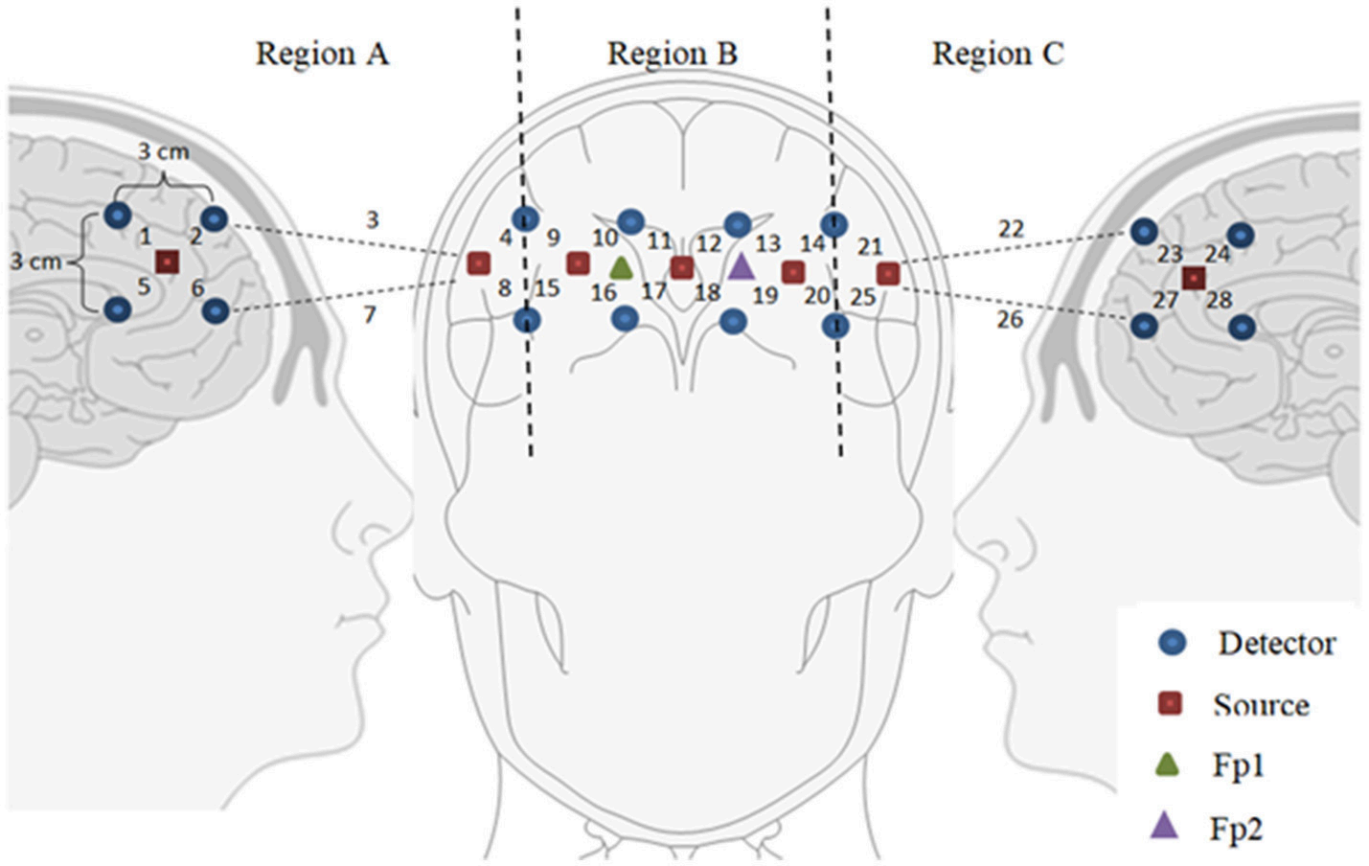
Partitioning the prefrontal cortex: Only a subregion showing the highest accuracy can be used for brain-computer interface purposes (for example, Region A was used by Khan and Hong, 2015).

This technique has a better chance of achieving high accuracy than the previous scheme. In this case, the brain region on which optodes are placed is divided into 2–3 smaller sub-regions (Scarpa et al., 2013; Ichikawa et al., 2014; Aghajani et al., 2017; Ge et al., 2017; Zhang et al., 2017). During the training phase, the channels in each sub-region are averaged and their accuracies are computed offline. The brain sub-region showing the highest accuracy is selected for the subsequent testing phase. A study by Khan and Hong (2015) used a total of 28 channels in the prefrontal region, which was divided into three sub-regions; sub-region A (the right side, 8 channels), sub-region B (the middle part, 12 channels), and sub-region C (the left side, 8 channels). Their study showed that sub-region A was more active than B or C. This approach is more effective than universal averaging, as it can narrow down the activated brain region. Moreover, fewer inactive channels are used for classification, thus improving the classification accuracy. Figure 4 shows an example of partitioning the prefrontal cortex. It is important to note that if only active channels are used for averaging, the accuracy can be further improved.

#### *t*-value based channel selection

If a stimulation paradigm is known, the *t*-value between the measured hemodynamic response and the expected hemodynamic response caused by the given stimulation can be computed. A *t*-value-based channel selection approach means that only those channels showing positive *t*-values are used for further analyses. In this case, a threshold value for *t* in selecting active channels can be set.

In computing the expected/desired hemodynamic response (dHRF), a type of standard hemodynamics response function, named the canonical hemodynamic response function (cHRF), *h*(*k*), is used. The dHRF is calculated by convolving the cHRF with the known stimulation interval, *s*(*k*), as follows.

(2) $$\begin{array}{l} u\left(k\right)=\overset{k-1}{\underset{n=0}{\sum }}h\left(n\right)s\left(k-n\right), \end{array}$$

where *u*(*k*) is the dHRF and the stimulation interval *s*(*k*) is defined as

(3) $$\begin{array}{l} s\left(k\right)=\{\begin{array}{c} 0,if\;k\in rest\\ 1,if\;k\in task \end{array}, \end{array}$$

where *rest* and *task* stand for the rest and task periods, respectively. The cHRF, *h*(*k*), is generated as a linear combination of two (or three) Gamma variant functions. If the rise of HbO upon brain stimulation and its undershoot afterward are considered, the cHRF is generated by two gamma functions as follows.

(4) $$\begin{array}{l} h\left(k\right)=\alpha_{1}\left[\frac{{\left(k/\tau_{1}\right)}^{\left(\phi_{1}-1\right)}e^{-\left(k/\tau_{1}\right)}}{\tau_{1}\left(\phi_{1}-1\right)!}-\alpha_{2}\frac{{\left(k/\tau_{2}\right)}^{\left(\phi_{2}-1\right)}e^{-\left(k/\tau_{2}\right)}}{\tau_{2}\left(\phi_{2}-1\right)!}\right], \end{array}$$

where α_1_ is the amplitude, τ_*i*_ and ϕ_*i*_ (*i* = 1, 2) tune the shape and scale respectively, and α_2_ is the ratio of the peak to the undershoot. If, on the other hand, the initial dip of HbO together with the peak and the undershoot of HbO need to be modeled, three gamma functions can be used as follows (Shan et al., 2014).

(5) $$\begin{array}{l} h\left(k\right)=\overset{3}{\underset{i=1}{\sum }}\left(\alpha_{i}\frac{k^{\phi_{i}-1}\tau_{i}^{\phi_{i}}e^{-\tau_{i}k}}{\Gamma \left(\phi_{i}\right)}\right), \end{array}$$

where α_*i*_, τ_*i*_, and ϕ_*i*_ for *i* = 1, 2, and 3 are the amplitude, time to peak, and width at the half peak value of the initial dip, main hemodynamic response, and undershoot, respectively.

For obtaining *t*-values, a typical linear regression model using the desired HR can be formulated as follows (Santosa et al., 2013, 2014).

(6) $$\begin{array}{l} h_{j}^{s}\left(k\right)=\phi_{j}^{s}u(k\text{)}+\psi_{j}^{s}\cdot 1+\varepsilon_{j}^{s}, \end{array}$$

where the superscript *s* denotes the stimulation number, *u*(*k*) ∈ *R*^*N*×1^ is the modeled hemodynamic response in (2), ϕ is an unknown coefficient that indicates the activity strength of the modeled hemodynamic response, ψ is a coefficient to compensate for baseline drift of the signal, **1** ∈ *R*^*N*×1^is a column vector of ones for correcting the baseline, and ε ∈ *R*^*N*×1^ is the error term in the regression model. The coefficient $\phi_{j}^{s}$ is estimated as $\phi_{j}^{s}$ using a recursive least squares algorithm (Ye et al., 2009).

The idea is to test the null hypothesis that the estimated parameter $\phi_{j}^{s}$ is equal to zero. If $\phi_{j}^{s}$ is positive, the specific activation is assumed to be active, and if it is negative, the specific activation is not active at the *j*-th channel, for which the *t*-value test has been used. The *t*-value is computed using the formula

(7) $$\begin{array}{l} t_{j}^{s}=\frac{{\hat{\phi }}_{j}^{s}}{SE\left({\hat{\phi }}_{j}^{s}\right)}, \end{array}$$

where *SE* is the standard error of the estimated coefficient. Two criteria to assess the selection reliability of a particular activation can be used: $t_{j}^{s}>$ 0 and $p_{j}^{s}<$ α_c_, where *p* denotes the *p*-value and α_c_ is the confidence interval. Alternatively, this could be done by checking $t_{j}^{s}$ > *t*_crt_, where *t*_crt_ denotes the critical *t*-value that depends on the degree of freedom (which is *N* −1). In the literature, the *t-*value based method is widely used for ROI selection, and also for brain imaging, in which the criterion is used to locate the activated brain areas (Hu et al., 2010; Al-Shargie et al., 2016; Li et al., 2017).

#### Baseline correction

Instead of computing the *t*-values for individual channels, the baseline during the rest period can be used as a criterion in selecting channels for further analysis. In this case, the maximum value during the task period and that of the rest period are compared. If the difference is positive, the channel is considered active (Hu et al., 2013). For example, the following criterion was used for channel selection by Khan and Hong (2017):

(8) $$\begin{array}{l} \delta p_{\text{trial}}\left(j\right)=\max\left(HbO_{\text{trial}}\left(k_{3}:k_{4},j\right)\right)-\max\left(HbO_{\text{rest}}\left(k_{1}:k_{2},j\right)\right), \end{array}$$

where δ*p*(*j*) represents the difference of the peak during the specified interval from that of the rest period, *j* is the channel number, *k*_1_ and *k*_2_ represent the interval for the rest period, and *k*_3_ and *k*_4_ represent the interval for the trial period. If δ*p*(*j*) is positive, the channel is selected as active; otherwise, the channel is discarded. Thus, this criterion reduces the computational time. For patients who might not be able to keep their heads steady during the acquisition of brain data, computation time becomes important. However, this criterion may result in higher variability throughout the BCI process. Due to this drawback, the baseline correction approach is seldom used. Therefore, it is better to track brain activity on each task to determine the correct brain regions.

#### Vector-phase analysis

This is a relatively new scheme in comparison to the methods described above. Since the vector phase plane is made of HbO and HbR, this method is available only for fNIRS-based BCIs; that is, this method is not applicable to EEG or fMRI. The term (i.e., phase-based analysis) does exist in EEG analysis, but applies to amplitude and phase analyses. The real component in the EEG vector plane is the original band-passed signal, and the imaginary component is the Hilbert transform of the signal, which corresponds to a 90° rotation of the real component (Foster et al., 2016). Therefore, the approach used for EEG has no relation with vector phase analysis as used for fNIRS.

**Figure 5:**
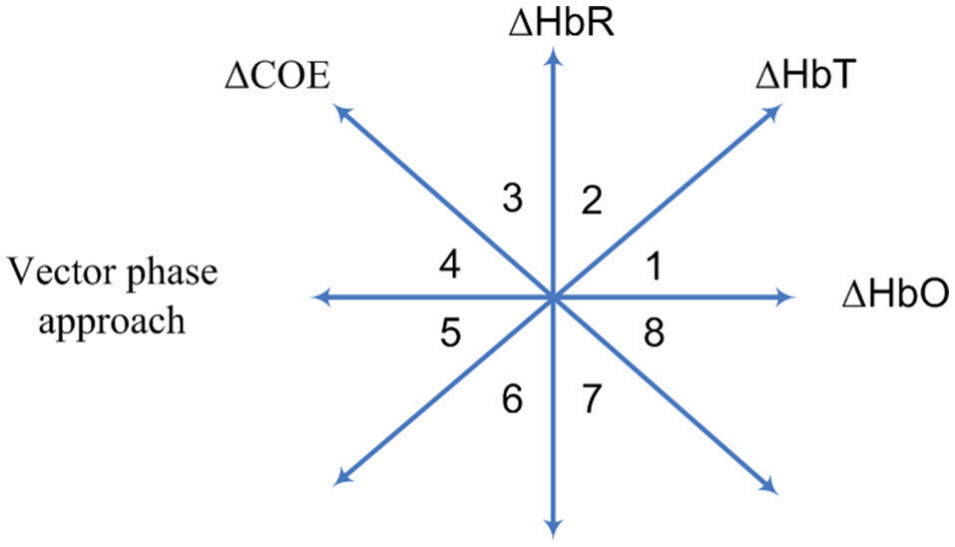
Vector-phase diagram proposed by Kato (2003).

Current research indicates that this method is suitable for hemodynamics-based systems for determination of the correct channels. The method was initially proposed for evaluating hemodynamics (Yoshino and Kato, 2012; Sano et al., 2013; Yoshino et al., 2013; Oka et al., 2015). Moreover, it is capable of predicting the trajectory of hemodynamic responses, which can be used for correct channel estimation. Furthermore, the detection of initial dips has been done by using vector-based phase analysis with a threshold circle as a decision criterion (Hong and Naseer, 2016), in which two independent vectors defined by oxy- and deoxy-hemoglobin (Δ*HbO* and Δ*HbR*) signals are orthogonally positioned. In addition to Δ*HbO* and Δ*HbR*, two other components can be defined as follows:

(9) $$\begin{array}{l} \Delta \;HbT=\frac{1}{\sqrt{2}}\left(\Delta \;HbO+\Delta \;HbR\right), \end{array}$$

(10) $$\begin{array}{l} \Delta \;COE=\frac{1}{\sqrt{2}}\left(\Delta HbR-\Delta HbO\right), \end{array}$$

where Δ*HbT* indicates the total hemoglobin concentration change and Δ*COE* denotes the cerebral oxygen change. Alternatively, these two components can be obtained by rotating the vector coordinate plane defined by Δ*HbO* and Δ*HbR* by 45° counterclockwise (see Figure 5). The magnitude and phase of a vector, *p*, in the plane can now be calculated as follows:

(11) $$\begin{array}{l} |p|=\sqrt{\Delta \;HbO^{2}+\Delta \;HbR^{2}}, \end{array}$$

(12) $$\begin{array}{l} ∠p=\tan^{-1}\left(\frac{\Delta \;HbR}{\Delta \;HbO}\right)=\tan^{-1}\left(\frac{\Delta \;COE}{\Delta \;HbT}\right)+45^{o}. \end{array}$$

The ratio of Δ*COE* and Δ*HbT* in (12) defines the degree of oxygen exchange, since Δ*COE* represents the oxygen exchange in the blood vessels and thus represents neuronal activity. Using the four indices, the phase plane is divided into eight phases (see Figure 5). The description of each phase is given in Table 2.

**Table 2:** Vector phases for initial dip and hemodynamics (Hong and Naseer, 2016).

**Phases**	**ΔHbO and ΔHbR states**	**ΔHbT and ΔCOE states**	**Conclusion**
1	Both positive ΔHbO > ΔHbR	ΔHbT is positive ΔCOE is negative	Initial dip phase
2	Both positive ΔHbO < ΔHbR	Both positive ΔHbT > ΔCOE	
3	ΔHbO is negative ΔHbR is positive	Both positive ΔHbT < ΔCOE	
4	ΔHbO is negative ΔHbR is positive	ΔHbT is negative ΔCOE is positive	
5	Both negative ΔHbO < ΔHbR	ΔHbT is negative ΔCOE is positive	
6	Both negative ΔHbO > ΔHbR	ΔHbT is positive ΔCOE is negative	Hemodynamic phase
7	ΔHbO is positive ΔHbR is negative	Both negative ΔHbT > ΔCOE	
8	ΔHbO is positive ΔHbR is negative	Both negative ΔHbT < ΔCOE	

The use of a threshold circle as a decision criterion, incorporated in the phase plane analysis, helps to minimize possible misidentifications of initial dips. The radius of the threshold circle for each channel is determined by detecting (i) the peak value of (11), or (ii) max(Δ*HbO*), or (iii) max(Δ*HbR*) during the rest state. The initial dip occurrence is concluded when the trajectories of (11) break out the threshold circle while its phase lies within the dip phases (i.e., phases 1–5). For hemodynamics, the activity can be considered concluded if the trajectory crosses the threshold circle in phases 6–8.

The key aspect of vector-phase analysis is the ability to detect the neural activity before the occurrence of hemodynamics. If we are able to tag the locations of individual brain signals, multiple brain signals can be detected as well. In this case, the addition of another threshold circle based on peak values of HbO may be useful to distinguish multiple brain activities using the analysis.

### Hardware configuration

For fNIRS-BCI, the majority of studies have used a source-detector-pair configuration, in which a source and a detector are separated at a distance of 3~5 cm for signal recording. Recently, a dense optode configuration has emerged in the literature as a potential tool for a condensed brain imaging. The methods for selecting the region of interest based on hardware cofiguration are discussed below:

#### Conventional configuration

Several different emitter-detector configurations have been used for fNIRS to record brain activities. The emitter-detector distance is usually kept within a specific range. To measure hemodynamic responses, an emitter-detector separation at around 3 cm is used. A separation of less than 1 cm might contain only skin-layer contribution, whereas that of more than 5 cm might result in weak signal detection. For the prefrontal cortex, 3 emitters and 8 detectors combination is usually used to record the brain activity for BCI (Naseer and Hong, 2015a). Mostly, for this configuration, channel averaging is used for selecting the region of interest.

#### Bundled-optode-based selection

The bundled-optode approach is a recent method of determining a precise brain region, which is currently being pursued for faster fNIRS-based brain imaging. This methodology involves spatially resolved spectroscopy (SRS) (Boas et al., 2014). The SRS approach was initially used to measure optical properties of tissues (Hunter et al., 2002; Kek et al., 2008). In this method, one emitter and at least two associated detectors are used, which means that at least two channels are required. The absorption coefficient can then be computed based on the absorbance gradient with respect to the emitter-detector distance. In this case, the NIRS optodes are grouped together to form a bundle. First, the brain location is determined using the International 10–20 system of electrode placement. The optode bundle is then placed on the activated brain region determine which portion is most active (Nguyen and Hong, 2016; Nguyen et al., 2016). In comparison to the optode placement method discussed in section Conventional Configuration, the bundled optode method is more spatially accurate as it removes the skin and skull absorption from the detected signal. Also, all the cortices of a patient may not be able to generate the desired brain activity for BCI. The conventional BCI system is not spatially precise in identifying the desired brain region (or channels) for a command generation. The bundled-optode scheme is able to distinguish the multiple tasks in a small brain region and it is spatially more accurate in identifying the designated brain. Therefore, it can contribute better in locating the activated brain region of a patient for BCI in comparison to the scheme that uses a few channels. Although the current setup may be bulky but, in the future, a micro-array type fNIRS can be developed.

**Figure 6:**
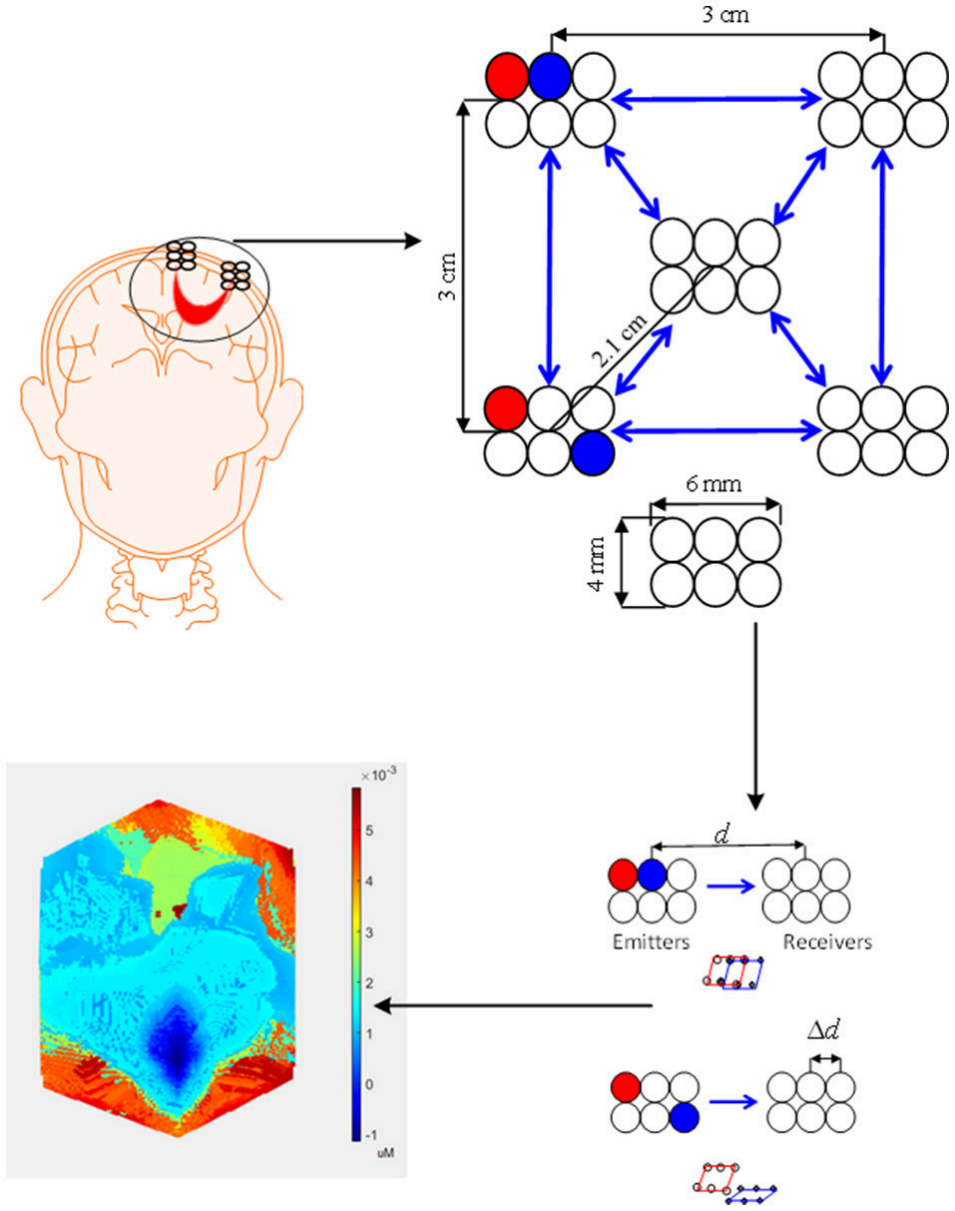
Bundled optode scheme: A schematic of densely configured fNIRS probes for deep brain imaging.

Figure 6 shows the configuration used for the bundled-optode-based scheme. The figure illustrates the concept of a bundled-optode configuration for the detection of a brain activity in a local brain region. The circles indicate emitters/detectors while the blue arrows illustrate the direction of light from emitters to detectors. The brain activity of each channel is detected as the mean values of HbX (i.e., HbO and HbR) that are encoded in to make the 3D color map. The *t*- or *p*-values can also be used for the reconstruction of the 3D image. Further research on this approach is needed, as it has a high potential of detecting a precise brain region of interest for each patient.

## Feature extraction and classification criteria

For generation of proper commands, the identification of correct features is essential. For use with patients this is even more crucial, as the use of poor features for classification may result in a significant drop in accuracy. The signal mean, signal peak, signal slope, latency, skewness, kurtosis, and power spectrum density are the most widely used features for both EEG and fNIRS (Lotte et al., 2007; Naseer and Hong, 2015a,b). Usually the features are categorized as temporal, spatial and spatio-temporal based on their characteristics (Robinson et al., 2016). The temporal features are evaluated using a specific time window for given data. The spatial features contain frequency-related information about the data in a specific time window. Increasing the number of features in a classifier increases the processing time. Typically, in fNIRS, a command for an active task is generated using a 2–7-s window. Thus, the extraction of features is directly dependent on the types of signals recorded.

### Feature extraction

In the fNIRS literature, various features have been reported for various types of tasks. In this section, we discuss the features most commonly used for an fNIRS-BCI.

#### fNIRS-based feature extraction

Many features can be estimated based on the HbO and HbR activity in a patient's brain. Among these features, the mean, peak, and slope are most commonly used (Hwang et al., 2014). A predetermined window is required to estimate these features (Gateau et al., 2015). Research has examined various sizes of windows, but a 2–7-s window from the onset time seems to show the best outcome for a 10-s task (Naseer and Hong, 2013).

##### Signal mean

The signal means of Δ*HbO* and Δ*HbR* are calculated as follows.

(13) $$\begin{array}{l} \mu_{w}=\frac{1}{N_{w}}\overset{k_{2}}{\underset{k=k_{1}}{\sum }}\Delta HbX\left(k\right), \end{array}$$

where the subscript *w* denotes “window,” μ_*w*_ is the mean value for a given window, *k*_1_ and *k*_2_ denote the start and end time of the window, *N*_*w*_ is the number of observations in the window, and Δ*HbX* represents the HbO or HbR data. Most fNIRS-based BCIs include the signal mean as a feature for classification (Hwang et al., 2016; Noori et al., 2017).

##### Signal slope

There are two ways to calculate the signal slope. The first method is to directly obtain the difference of the values at the start and end points of a predetermined interval (for example, from 2–7 s from the onset time of a stimulus, or the entire stimulus period plus a portion of the subsequent rest period) and to use them to compute the slope (Shin and Jeong, 2014). The second method uses a curve-fitting approach to fit a line to the hemodynamic signal for the predetermined interval. The first method is more widely used, but the second method may be preferable (Weyand et al., 2015b).

##### Signal peak

This feature is the peak value of the signal in a given window. Some studies have reported that peak values worked best in fNIRS (Stangl et al., 2013; Shin and Jeong, 2014).

##### Signal minimum

This feature is the minimum value of the signal in a given window. Most commonly this feature has been used to identify an initial dip for a 2-s window. To the best of our knowledge, only three studies have so far used this feature for fNIRS-BCI (Khan and Hong, 2017; Li et al., 2017; Zafar and Hong, 2017).

##### Skewness and kurtosis

The skewness is computed as follows:

(14) $$\begin{array}{l} skew_{w}=\frac{E_{x}{\left(\Delta HbX_{w}-\mu_{w}\right)}^{3}}{\sigma^{3}}, \end{array}$$

where *skew* is the skewness, σ is the standard deviation of Δ*HbX* for the given window, and *E*_*x*_ is the expectation of Δ* HbX*. The kurtosis is computed as follows.

(15) $$\begin{array}{l} kurt_{w}=\frac{E_{x}{\left(\Delta HbX_{w}-\mu_{w}\right)}^{4}}{\sigma^{4}}, \end{array}$$

where *kurt* is the kurtosis ofΔ*HbX*. Both skewness and kurtosis have been reported to work moderately well for fNIRS (Hong and Santosa, 2016; Hwang et al., 2016).

##### Number and sum of peaks

The number of peaks is calculated by measuring the local maxima of the ΔHbO signal in a single time window. The *findpeaks* function available in MATLAB can be used to measure the number of peaks (either online or offline). The sum of peaks is obtained by summing the local maxima in a given window (Khan and Hong, 2015).

##### Others

A few other features such as the variance (Holper and Wolf, 2011), root mean square (Tai and Chau, 2009; Watanabe et al., 2016), standard deviation (Abibullaev et al., 2011) and median (Shin and Jeong, 2014) have also been reported to work for fNIRS. The fast optical response can be used as a feature (Hu et al., 2013). The conversion of optical signals into HbO and HbR can give more information about the brain activity.

#### Feature extraction for hybrid modalities

EEG and fNIRS are the two main BCI modalities where mobility is required, and recent research has shown that these two systems can be integrated to improve the BCI performance. Most commonly, EEG features are extracted from frequency bands that are related to specific brain activity. For current control applications, event-related desynchronization/event-related synchronization (ERD/ERS) based features are combined with fNIRS to improve the system performance. There is a study that combined EEG and fNIRS for a SSVEP task (Tomita et al., 2014). In our opinion, ERD/ERS based activities can be more useful for patients than SSVEP, as they can be generated deliberately. EEG feature extraction schemes are now briefly discussed.

##### Power spectrum density

The power spectral density (PSD) describes the strength of a signal as a function of frequency. For nonparametric methods, the autocorrelation sequence for a given set of data is first estimated. The PSD is then calculated by Fourier transforming the estimated autocorrelation sequence. One way of doing this is using the Welch's method. The method is applied to a moving window, producing a modified periodogram. The power spectrum density is expressed as follows:

(16) $$\begin{array}{l} P_{f}^{s}=\frac{1}{N}\overset{N}{\underset{n=1}{\sum }}{\left|{x_{n}}_{s}e^{-j2\pi fn}\right|}^{2}, \end{array}$$

where $P_{f}^{s}$ denotes the power of the *f*-th frequency band in the *s*-th data sequence, *x*_*ns*_ is the magnitude of the *s*-th sequence, and *N* is the number of samples. Several EEG based control studies have used this method for evaluation of features (Tanaka et al., 2005; Carlson and Millan, 2013). Moreover, most hybrid EEG-fNIRS BCI studies have used the power spectrum as a classification feature (Putze et al., 2014; Tomita et al., 2014).

##### Logarithmic band power

As the name suggests, this feature is estimated using the logarithms of the power of different bands of EEG data. To estimate this feature, first the power value of each frequency band is estimated (see Equation 16). The logarithm of the signal power is taken to estimate the highest value in the band:

(17) $$\begin{array}{l} LP_{f}=\log\left(P_{f}\right), \end{array}$$

where *LP*_*f*_ is the logarithmic power of the signal. This approach is used in some wheelchair control studies (Tsui et al., 2011; Lee et al., 2015).

##### Common spatial patterns

Common spatial patterns (CSP) for EEG feature extraction and classification are used to project the multi-channel EEG data into a low-dimensional spatial subspace with a projection matrix of which each row consists of weights for channels. This transformation can maximize the variance of two-class signal matrices. The CSP method is based on simultaneous diagonalization of covariance matrices for the two classes.

To calculate the CSP features, first a sample covariance matrix for a trial is calculated as follows:

(18) $$\begin{array}{l} R=\frac{XX^{T}}{tr\left(XX^{T}\right)}, \end{array}$$

where *X* is the sample data, *tr(X)* denotes the trace of a matrix, and *T* denotes the transpose of a matrix. The composite spatial covariance is estimated as follows:

(19) $$\begin{array}{l} {\bar{R}}_{1}+{\bar{R}}_{2}=UAU^{T}, \end{array}$$

where *U* denotes the matrix of eigenvectors, and *A* denotes the diagonal matrix of corresponding eigenvalues. The full projection matrix is then formed as follows:

(20) $$\begin{array}{l} W=B^{T}\sqrt{A^{-1}}U^{T}, \end{array}$$

where *B* denotes the matrix of Eigen vectors for the whitened spatial covariance matrix. The eigenvalues and eigenvectors are sorted in descending order, from first to last:

(21) $$\begin{array}{l} Z=W^{T}X. \end{array}$$

A 2-dimensional feature is then constructed from the variance of the rows of *Z*:

(22) $$\begin{array}{l} f_{q}=\log\left(\frac{var\left(Z_{q}\right)}{\overset{2}{\underset{i=1}{\sum }}var\left(Z_{i}\right)}\right), \end{array}$$

where *Z*_*q*_is the *q*-th row of vector *Z*. The CSP method of feature extraction has been adopted for wheelchair control using both active and reactive tasks (Li et al., 2013b, 2014; Cao et al., 2014; Buccino et al., 2016; Zhang et al., 2016; Ge et al., 2017; Shin et al., 2017).

##### Others

There have been a few studies that have also used the time frequency phase (Yin et al., 2015b) and the coefficients of a wavelet transform (Li et al., 2017) as features for EEG, which were combined with fNIRS for hybridization. In an example of hybrid EEG-fNIRS, Blokland et al. (2014) used band power and logistic regression coefficients as features with a 0–15-s window for EEG and a 3–18-s window for fNIRS for tetraplegia patients.

### Classifiers for hybrid EEG-fNIRS

Classification techniques are used to identify different brain signals that are generated by the user. These identified signals are then translated into control commands for application interface purposes. In most existing fNIRS-BCIs, identification is performed by using classification techniques to discriminate various brain signals based on appropriate features. Classification algorithms, calibrated by supervised learning during a training phase, can detect brain-signal patterns during the testing stage. Here, we will discuss only the classifiers that are most commonly used for hybrid EEG-fNIRS.

#### Linear discriminant analysis

Linear discriminant analysis (LDA) is the most commonly used classification method in fNIRS and hybrid EEG-fNIRS studies. It utilizes discriminant hyperplane(s) to separate data representing two or more classes. Because of its simplicity and low computational requirements, it is highly suitable for online BCI systems. In LDA, the separating hyperplane is found by seeking a data projection that maximizes the distance between the means of two or more classes and minimizes the interclass variances. LDA assumes a normal data distribution along with equal covariance matrices for both classes.

The optimal projection matrix *V* for LDA that maximizes the corresponding Fisher's criterion is given as follows:

(23) $$\begin{array}{l} J\left(V\right)=\frac{\det\left(V^{T}S_{B}V\right)}{\det\left(V^{T}S_{W}V\right)}, \end{array}$$

where *S*_*B*_ and *S*_*W*_ are the between-class scatter matrix and the within-class scatter matrix, respectively, which are defined as follows:

(24) $$\begin{array}{l} S_{W}=\overset{m}{\underset{i=1}{\sum }}\underset{x_{k}\in class\;i}{\sum }\left(x-\mu_{i}\right){\left(x-\mu_{i}\right)}^{T} \end{array}$$

(25) $$\begin{array}{l} S_{B}=\overset{m}{\underset{i=1}{\sum }}n_{i}\left(\mu_{i}-\mu \right){\left(\mu_{i}-\mu \right)}^{T}. \end{array}$$

Here, *x* ∈ *R*^2^ denotes the samples, μ_*i*_ is the sample mean of class *i*, and μ is the total mean of all the samples, *m* is the total number of classes, *n*_*i*_ is the number of samples of class *i*, and *n* is the total number of samples. Equation (27) is treated as an eigenvalue problem to obtain the optimal vector *V* that corresponds to the largest eigenvalues. Many fNIRS and hybrid EEG-fNIRS studies have successfully used LDA for BCIs (Luu and Chau, 2009; Bauernfeind et al., 2011; Fazli et al., 2012; Moghimi et al., 2012; Khan et al., 2014; Lee et al., 2015; Ahn et al., 2016; Buccino et al., 2016; Shin et al., 2017).

#### Support vector machines

The support vector machine (SVM) is a very popular pattern recognition technique for brain signal classification. It has been used in various fNIRS studies (Sitaram et al., 2007; Cui et al., 2010; Tanaka and Katura, 2011; Putze et al., 2014; Koo et al., 2015). It is a supervised classifier that can define two or more classes by determining the maximum class separation, known as the “maximum margin hyperplane.” The algorithm does this by mapping the input data to a feature space that can be divided using linear or non-linear decision boundaries, depending on the kernel. The SVM classifier tries to maximize the distance between the separating hyperplane and the nearest training point(s) (the so-called support vectors). The separating hyperplane in the 2D feature space is given by the equation

(26) $$\begin{array}{l} f\left(x\right)=r\cdot x+b_{1}, \end{array}$$

where *r, x* ∈ *R*^2^ and *b* ∈ *R*^1^. The optimal solution r^*^ that maximizes the distance between the hyperplane and the nearest training point(s) can be obtained by minimizing the cost function

(27) $$\begin{array}{l} L\left(r,\xi \right)=\frac{1}{2}{\left||r|\right|}^{2}+C\cdot \sum_{n=1}^{Z}\xi_{n}, \end{array}$$

while satisfying the constraints

(28) $$\begin{array}{r} \left(x_{n}.r+b_{1}\right)\geq 1-\xi_{n}\;for\;y_{n}=+1,\\ \left(x_{n}.r+b_{1}\right)\geq -1+\xi_{n}\;for\;y_{n}=-1\\ \xi_{n}\geq 0\;\forall n, \end{array}$$

where ||*r*||^2^ = *r*^*T*^*r*, *C* is a positive regularization parameter chosen by the user (a large value of *C* corresponds to a high penalty for classification errors), ξ_*n*_ is a measure of training error, *z* is the number of misclassified samples, and *y*_*n*_ is the class label (+1 or −1 in the case of binary classification) for the *n*-th sample.

The radial basis function (RBF) kernel is widely used, as it allows complex separation surfaces requiring a reduced number of hyper-parameters to tune. The hyper-parameters for this SVM give an upper bound on the fraction of margin errors and a lower bound on the fraction of support vectors. For a multi-class problem, the data are subdivided into several binary class problems and used in a one-against-one approach. Thus, for an *m*-class problem, *m*(*m*−1)/2 machines are trained.

#### Extreme learning machine

The extreme learning machine (ELM) is a learning algorithm in which single-hidden-layer feedforward neural networks are used for classification and regression. The ELM training algorithm can adaptively set the number of hidden layer nodes and randomly assigns the input weights and hidden layer biases. The output layer weights are obtained by the least squares method, and the whole learning process is completed in one calculation stage without iteration (Deng et al., 2010). The ELM for *N* arbitrary distinct samples (*x*_*i*_, *l*_*i*_), where *x*_*i*_ = [*x*_*i*1_, *x*_*i*2_, … *x*_*i*n_]^*T*^ ∈ *R*^*n*^ and *l*_*i*_ = [*l*_*i*1_, *l*_*i*2_, … *l*_*i*m_]^*T*^ ∈ *R*^*m*^, and (*x*_*i*_, *l*_*i*_) ∈ *R*^*n*^ x *R*^*m*^ (*i*-1,2,…,*N*), for a standard single-layer feedforward neural network with *N*_H_ hidden nodes and activation function *g*(*x*), is given as follows:

(29) $$\begin{array}{l} \overset{N}{\underset{i=1}{\sum }}\beta_{i}g_{i}\left(x_{d}\right)=\overset{N}{\underset{i=1}{\sum }}\beta_{i}g_{i}\left(a_{i}.x_{d}+b_{i}\right)=o_{d},d=1,2,\ldots ,N, \end{array}$$

where *a*_*i*_ is the weight vector connecting the *i*-th hidden neuron to the input, and *b*_*i*_ is the threshold of the *i*-th hidden neuron, β_*i*_ is the weight vector connecting the *i*-th hidden neuron to the output, and *o*_*d*_ is the *d*-th output vector of single layer feed forward neural network. The above equation can be written as follows:

(30) $$\begin{array}{lll} H\beta =O,\\ {\left[\begin{array}{ccc} f\left(a_{1}.x_{1}+b_{1}\right)\cdots f\left(a_{N}.x_{N}+b_{N}\right)\\ \vdots & \cdots & \vdots \\ f\left(a_{1}.x_{N}+b_{1}\right) & \cdots & f\left(a_{N}.x_{N}+b_{N}\right) \end{array}\right]}_{N\times N},\\ \beta & = & {\left[\begin{array}{c} \beta_{1}^{T}\\ \vdots \\ \beta_{N}^{T} \end{array}\right]}_{N\times m},O={\left[\begin{array}{c} o_{1}^{T}\\ \vdots \\ o_{N}^{T} \end{array}\right]}_{N\times m} \end{array}$$

where *H*(*a*_1_, *a*_2_, …, *a*_N_, *b*_1_, *b*_2_, …, *b*_N_, *x*_1_, *x*_2_, …, *x*_N_) is called the hidden layer output matrix of the neural network. After the input weights and hidden layers biases are determined in accordance with the random assignment, the input samples are used to obtain the hidden layer output matrix *H*. A least squares solution for the above equation can be found as follows:

(31) $$\begin{array}{l} \overset{\wedge }{\beta }=H^{†}O, \end{array}$$

where *H* represents the Moore-Penrose generalized inverse of the hidden layer output matrix *H*. The optimal solution $\hat{\beta }$ controls the minimal training error gain of the algorithm. A few fNIRS studies have used ELM for BCI (Yin et al., 2015a,b).

#### Vector phase analysis as a classifier

**Figure 7:**
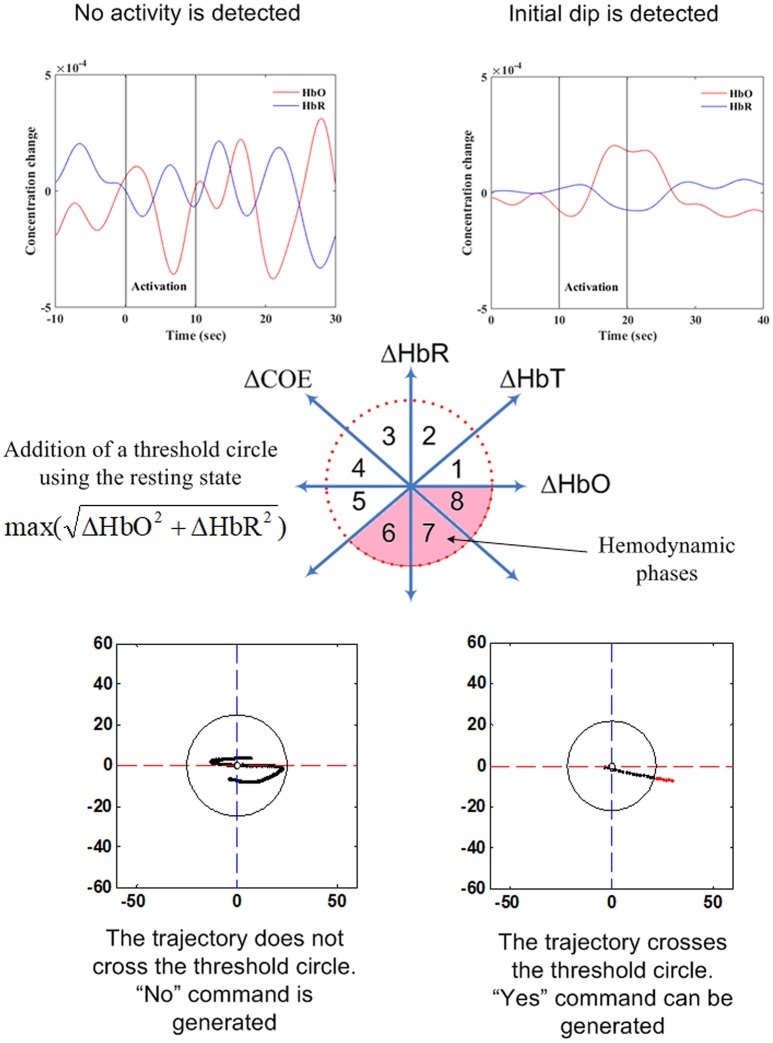
Illustration of vector-phase analysis for two choice decoding.

As discussed earlier, vector phase analysis can be used for identification of brain regions (see the Vector-Phase Analysis section). It has applications in classification as well. To the best of our knowledge, only one study has used this classifier to decode two different brain states (Zafar and Hong, 2017). The current limitation in this method is that it has been used only to distinguish between resting and activity states. Further research in this area is needed to improve the usability of the method for decoding multiple tasks. Figure 7 shows the strategy adopted by vector phase analysis for decoding two choices.

Deep learning algorithms can also be a potential candidate for classification of brain images. The conventional classifiers may not be effective for identification of neuro-plasticity from brain images. So far, only one fNIRS study has used deep learning approach for BCI (Trakoolwilaiwan et al., 2017). As described in the study, a Convolutional Neural Network (CNN) can be effective for classification of multiple brain activities from a brain image. Just like the extreme learning machine, a CNN is comprised of one or more layers that are convolutional followed by one or more fully connected layers. The architecture of a CNN is designed to take advantage of the 2D structure of an input image that can be beneficial to detect an activity from a brain map. CNN can be trained with a few parameters and thus the command generation for BCI control may not be increased.

**Figure 8:**
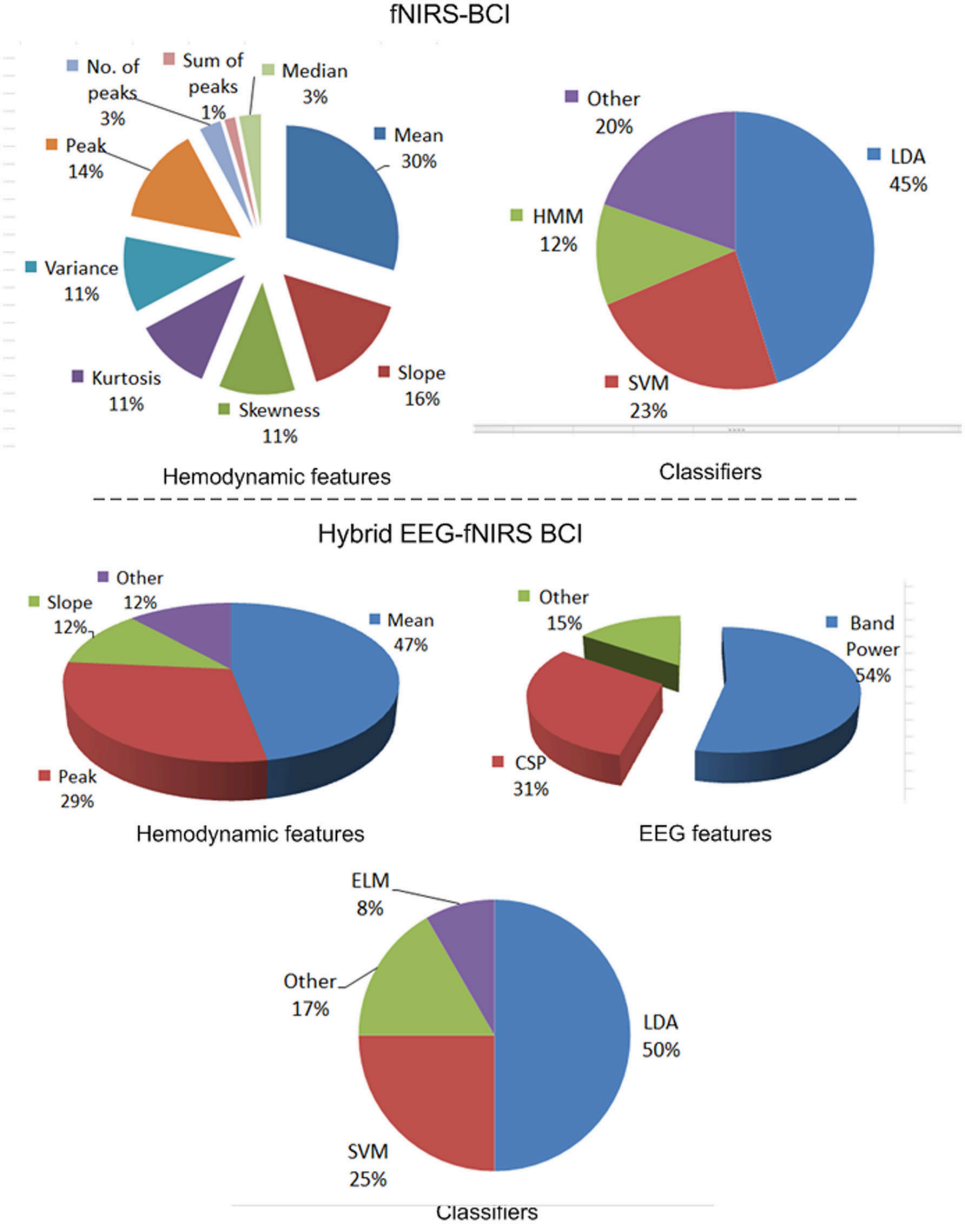
Features and classifiers used in fNIRS and hybrid EEG-fNIRS studies (64 fNIRS-BCI papers and 14 hybrid EEG-fNIRS papers from 2010 to 2017).

Figure 8 shows distributions of the features and classifiers that have been used during 2010–2017 for fNIRS. The lower part of the figure shows information on hybrid EEG-fNIRS features and classifiers used to generate multiple commands.

## Device interfaces

The purpose of a BCI is not achieved if a final interface with a machine is not provided for communication with a patient. It can be difficult for a patient to generate multiple commands needed to operate a complex system. A simplified and easy interface is required for a BCI to be taken out of the laboratory environment. The BCI should be designed in accordance with the number of commands that a patient is able to generate. Even if a reactive BCI may be able to generate multiple commands, it may not be a viable option for real life. It is obvious that if the wrong brain region is selected for activity detection, a proper signal for BCI cannot be generated. Most fNIRS studies have not yet provided a device interface that can prove its utility for patients. To discuss the major role of device interfaces in fNIRS and fNIRS-based hybrid BCI, we categorize the existing studies based on the types of applications.

### Choice selection

A great majority of fNIRS studies for BCI have focused on decoding only two commands. A list of these studies was provided in Table 3. It can be clearly seen from the table that only a single study was able to implement their BCI for LIS patients. The remaining studies do not show any evidence of working with patients. The table also shows that the signal mean was used most often as a feature, with LDA for classification. Moreover, none of the studies was able to implement their BCI in real-time. Probably the inherent delay of the hemodynamic signals was a restricting factor; this problem is not easy to overcome, because implementation of the results for real-time applications may significantly reduce the accuracy. Even so, after examining the literature, we can say that fNIRS-based BCIs have the capability to decode two choices for patients using active paradigms. Further research with LIS patients as subjects can focus on finding optimal features that can be used to enhance the classification accuracy and to increase the number of commands available for control.

**Table 3:** fNIRS-BCI studies (2012–2017) that decoded brain activities from the prefrontal cortex.

**References**	**Type of subject**	**Brain region selection scheme**	**Analysis type**	**Task**	**Features**	**Classifier**	**Possible application**	**Number of commands**	**Classification accuracy (%)**
Hu et al., 2012	Healthy	Channel averaging	Online	Truth/lie	Absolute values of ΔHbO and ΔHbR	SVM	2 choice decoding	2	83.4
Power et al., 2012a	Healthy	Channel averaging	Offline	Mental arithmetic	Signal slope	LDA	2 choice selection	2	72.6
Power et al., 2012b	Healthy	Channel averaging	Offline	Mental arithmetic and mental singing	Signal slope of linear regressing line	LDA	Can be used for wheel chair control	3	56.2
Chan et al., 2012	Healthy	Channel averaging	Offline	Mental singing	Peak of ΔHbO and ΔHbR	HMM and ANN	2 choice decoding	2	55.7 for HMM and 63 for ANN
Abibullaev and An, 2012	Healthy	Single channel selection based on wavelet coefficients	Offline	Object rotation, letter padding and multiplication	Filter coefficients from wavelet transform	LDA and SVM	Applicable for wheelchair control	2 (can be used to generate 4 commands)	> 85 (LDA) > 90 (SVM)
Moghimi et al., 2012	Healthy	Channel averaging	Offline	Music listning	Mean and difference between signal and noise of ΔHbO and ΔHbR	LDA	2 choice decoding	2	71.9
Power and Chau, 2013	Duchenne muscular dystrophy patient	Individual channel used	Online	Mental arithmetic	Signal slope of ΔHbO and ΔHbR	LDA	2 choice decoding	2	71.1
Stangl et al., 2013	Healthy	Channel averaging	Online	Motor imagery, mental arithmetic	Amplitude of ΔHbO	LDA	2 choice decoding	2	65
Faress and Chau, 2013	Healthy	Individual channel used	Offline	Verbal fluency	Slope of HbO, HbR and HbT	LDA	2 choice decoding	2	86
Schudlo and Chau, 2014	Healthy	Individual channel used	Online	Mental arithmetic	Slope of ΔHbO, ΔHbR and ΔHbT	LDA	2 choice decoding	2	77.4
Naseer et al., 2014	Healthy	Channel averaging	Online	Mental arithmetic	Mean values of ΔHbO and ΔHbR	LDA and SVM	2 choice decoding	2	74.2 (LDA) 82.1 (SVM)
Hwang et al., 2014	Healthy	Channel averaging	Offline	Motor Imagery, mental singing, mental arithmetic, mental rotation and mental character writing	Mean values of HbO, HbR and HbT	LDA	2 choice decoding	2	> 70 (mental arithmetic and mental rotation)
Herff, 2014	Healthy	Individual channel used	Offline	n-back task for mental workload	Slope of HbO and HbR	LDA	Mental workload measurement	2	78
Khan and Hong, 2015	Healthy	Brain segmentation to identify precise location	Online	Active and drowsy state	Mean, peak and sum of pekas of ΔHbO	LDA and SVM	Drowsiness detection	2	83.1 (using LDA) 84.4 (using SVM)
Hong et al., 2015	Healthy	Channel averaging	Online	Motor imagery, mental arithmetic	Mean and slope of HbO	LDA	Can be used for wheelchair control	3	75.6
Naseer and Hong, 2015b	Healthy	Channel averaging	Online	Motor imagery and mental arithmetic	Mean and slope of HbO and HbR	LDA	4 choice selection (can be used for wheelchair control)	4	73.3
Bhutta et al., 2015	Healthy	Channel averaging	Online	Truth and lie	Signal mean and signal slope	LDA	2 choice decoding	2	86.5
Weyand et al., 2015a	Healthy	Individual channel used	Online	11 mental tasks	Changes in HbO, HbR and HbT	LDA	2 choice decoding	2	76.0
Yin et al., 2015a	Healthy	Individual channel	Online	Motor	Difference of HbO and HbR	ELM	Applicable to wheelchair control	3	>75
C Schudlo and Chau, 2015a	Healthy	Individual channel used	Online	Verbal fluency, Stroop and rest	Slope of HbO, HbR and HbT	LDA	Can be used for wheelchair control	3	71.7
Schudlo and Chau, 2015b	Healthy	Individual channel used	Offline	Verbal fluency, Stroop and rest	Slope of HbO, HbR and HbT	LDA	2 choice decoding	2	82.8
Weyand et al., 2015b	Healthy	Individual channel	Online	5 mental tasks	Temporal changes in HbO, HbR and HbT	LDA	2 choice decoding	2	76.6
Weyand and Chau, 2015	Healthy	Individual channel	Online	6 mental tasks	Temporal changes in HbO, HbR and HbT	LDA	Can be tested for wheelchair control	Upto 5	78.0 for 2 class 37.0 for 5 class
Durantin et al., 2016	Healthy	Averaging	Offline	Digit memorization	Peak of HbO and HbR	SVM	2 choice decoding	2	77.8%
Naseer et al., 2016a	Healthy	Averaging	Offline	Mental arithmetic	Mean, slope, variance, peak and kurtosis	LDA	2 choice decoding	2	93.0
Naseer et al., 2016b	Healthy	Averaging	Offline	Mental arithmetic	Mean, slope, variance, peak and kurtosis	LDA, QDA, KNN, Bayes, SVM and ANN	2 choice decoding	2	96.3
Zafar and Hong, 2017	Healthy	Averaging on specific channels	Offline	Mental arithmetic, mental counting and puzzle solving	Initial dip features (signal mean and signal minimum of ΔHbO)	Vector phase analysis and: LDA	2 choice decoding	2	57.5 for initial dip
Qureshi et al., 2017	Healthy	Averaging	Offline	Motor imagery and mental rotation	Coefficients of GLM	LDA	Can be used for wheelchair control	3	87.8
Chaudhary et al., 2017	Amyotrophic lateral sclerosis	Individual channel	Online	Mental listening task	Signal mean	SVM	Choice decoding	2	70.0

### Gait and balance control

Mihara et al. (2008) have reported that the cortical activation associated with postural adjustment of a patient results in a significant increase in HbO in the bilateral prefrontal cortex. These activations can be used to control a robotic interface for postural control and balancing of a locked-in patient (Khan et al., 2018). However, further research is needed in this domain to decode multiple postures for patients.

### Brain plasticity monitoring

fNIRS can serve as a tool for monitoring of neuroplasticity and functional recovery after brain injury. Patients with acute brain injury such as stroke can take months to recover from the injury (Jørgensen et al., 1999). fNIRS can be used for estimating the cortical plasticity of the patient's brain. However, deep brain imaging may be required to estimate different stages of brain recovery/plasticity of a patient. For these cases, deep learning algorithms like convolutional neural network can play a vital role in estimation of brain plasticity/recovery.

### Wheelchair control

In this category, we selected only fNIRS and hybrid EEG-fNIRS studies in which more than two commands were generated. The list of these studies is provided in Tables 3, 4. It can be seen from Table 3 that the accuracy of fNIRS-BCI is very low for 5-command decoding. However, the accuracy for four or more commands becomes higher if a hybrid EEG-fNIRS technique is used. Patients who suffer severely from motor disorders may not be able to generate many commands even with hybridization. However, an increase in the number of commands and enhancement in accuracy promises more safety and flexibility for patients. The main reason is that the commands generated using one brain signal acquisition modality can be verified with the other; thus, the accuracy is improved. Although wheelchair control has not yet been implemented using fNIRS alone, hybridization might achieve this objective with enhanced accuracy.

**Table 4:** Features and classifiers used for hybrid EEG-fNIRS.

**References**	**Type of subject**	**Analysis type**	**Task**	**NIRS features**	**EEG features**	**Classifier**	**Possible application**	**Number of commands**	**Classification accuracy (%)**
Fazli et al., 2012	Healthy	Offline	Motor tasks	Mean ΔHbO, ΔHbR and ΔHbT	Band power	LDA	Choice decoding	2	>90
Tomita et al., 2014	Healthy	Offline	SSVEP	First and second derivative of ΔHbO and ΔHbR	Band power	Joint classifier	Multiple choice selection and wheelchair control	2	>90
Khan et al., 2014**	Healthy	Online	Motor execution, mental counting and mental arithmetic	Mean values of ΔHbO and ΔHbR	Band power	LDA	Wheelchair control	4	> 80
Blokland et al., 2014	Tetraplegia patients	Offline	Motor task	Mean of HbO and HbR in 3~18 sec window	Band power	Linear logistic regression classifier	Choice decoding	2	Average accuracy >80
Putze et al., 2014	Healthy	Offline	Audio and video perception	Difference of mean of HbO and HbR	Band power	SVM	Choice decoding	2	Highest accuracy>90
Koo et al., 2015	Healthy	Online	Motor task	Threshold for HbO	CSP	SVM for EEG and threshold for fNIRS	Choice decoding	2	>85
Lee et al., 2015	Healthy	Online	Motor task	Mean amplitude of HbO and HbR	CSP and Logarithmic power	LDA	Choice decoding	2	>85
Yin et al., 2015b	Healthy	Online	Motor	Difference of HbO and HbR	Time-frequency Phase	ELM	Choice decoding	2	>89
Buccino et al., 2016	Healthy	Offline	Motor	Signal mean and Signal slope of HbO	CSP	LDA	Applicable to wheelchair control	4	>70
Ahn et al., 2016	Healthy	Online	Drowsiness	Amplitude of HbO and HbR	Band power	LDA	Sleep task	2	>75
Khan and Hong, 2017	Healthy	Online	Mental task	Initial dip and hemodynamic features (Mean, minimum and peak of HbO in 2 sec window)	Band power and Peak amplitude	LDA	Quadcopter control (More possibilities for wheelchair control)	8	>75
Li et al., 2017	Healthy	Online	Motor	Mean values of HbO and HbR in 2 sec window	Coefficient of wavelet transform	SVM	Choice decoding in 2 sec window	2	>90
Aghajani et al., 2017	Healthy	Online	Working memory	Peak, slope, standard deviation, skewness and kurtosis of HbO and HbR	Band power and phase locking value	SVM	Mental fatigue estimation	2	>80
Ge et al., 2017	Healthy	Offline	Motor	Hurst exponent	CSP	SVM	Choice decoding	2	>80

## What future direction to adopt?

From the information presented here, it can be deduced that we are still far from successfully implementing a BCI for a patient that works in real-time. However, the current research trends are headed in the right direction and the goal may not be impossible to achieve. There are certain issues that must be resolved first to allow more rapid progress toward achieving the goal.

The first requirement is to use patients instead of healthy subjects to validate a BCI method. Even if BCI schemes can be successfully implemented on healthy persons, it may be difficult to use the same methods for patients. Many studies suggest that high accuracy for patients should be possible, but the reality may be different (Chaudhary et al., 2017).

The second necessity is the selection of appropriate brain activity for LIS patients. Currently, we cannot say for sure which brain task is best suited for a patient. A healthy person can perform any reasonable task, but a patient requires a task from a number of options. Strong conclusions can only be drawn if large samples of patients are used to evaluate different types of brain tasks.

Another issue is the selection of a good brain region. As discussed, most studies have focused on averaging or selecting a single channel for BCI. A precisely localized brain location is required for high-quality detection of brain activity. In this context, further research on the bundled optode scheme using fNIRS is required to narrow down the optimal brain region for a patient. Moreover, deep brain imaging techniques may have a possibility of better discriminating brain signals than conventional methods (Nguyen and Hong, 2016; Nguyen et al., 2016).

Another point raised here is that most fNIRS studies have not physically implemented their BCI. There might be benefit in using additional external sensors (e.g., blood-pressure monitoring, respiration, and cardiac monitoring at the same time) to make the final decision to generate a command. Even for a very basic problem of Yes/No decoding (see section Choice Selection), an incorrectly generated command may lead to severe consequences in a real environment; especially for a locked-in patient who may not be able to respond to a misclassified decision. Therefore, improvement in this category is needed.

Most BCI studies have found that the output from hemodynamic or neuronal signals are sufficient for device control. However, the high accuracy sometimes occurs due to false detection of brain signals. For real-time control, this false positive detection may create a safety issue for LIS patients. Therefore, a better approach is to hybridize neuronal and hemodynamic signals. Current research on hybridization has only used system based on combinations of probability scores (Buccino et al., 2016). There is a need to develop new methods of integrating neuronal and hemodynamic deep brain activity.

One more issue in achieving device control is reliability of the brain signals from patients. It may be difficult for a patient to concentrate on the brain task for a long duration. Thus, the accuracy for controlling a machine will be reduced significantly. It will be better if a shared control is implemented to achieve better outcome for a patient. The shared control is to use an artificial intelligence technique combined with a BCI to improve the control performance. For example, in the case of a wheelchair control, a semi-autonomous control can be implemented. The brain signals are used to activate/deactivate the wheelchair whereas a motion planning algorithm can be implemented for the wheelchair to reach the destination. In this case, the patient can concentrate less on generating the brain activity for control, and this will reduce the stress and anxiety level of the patient.

**Figure 9:**
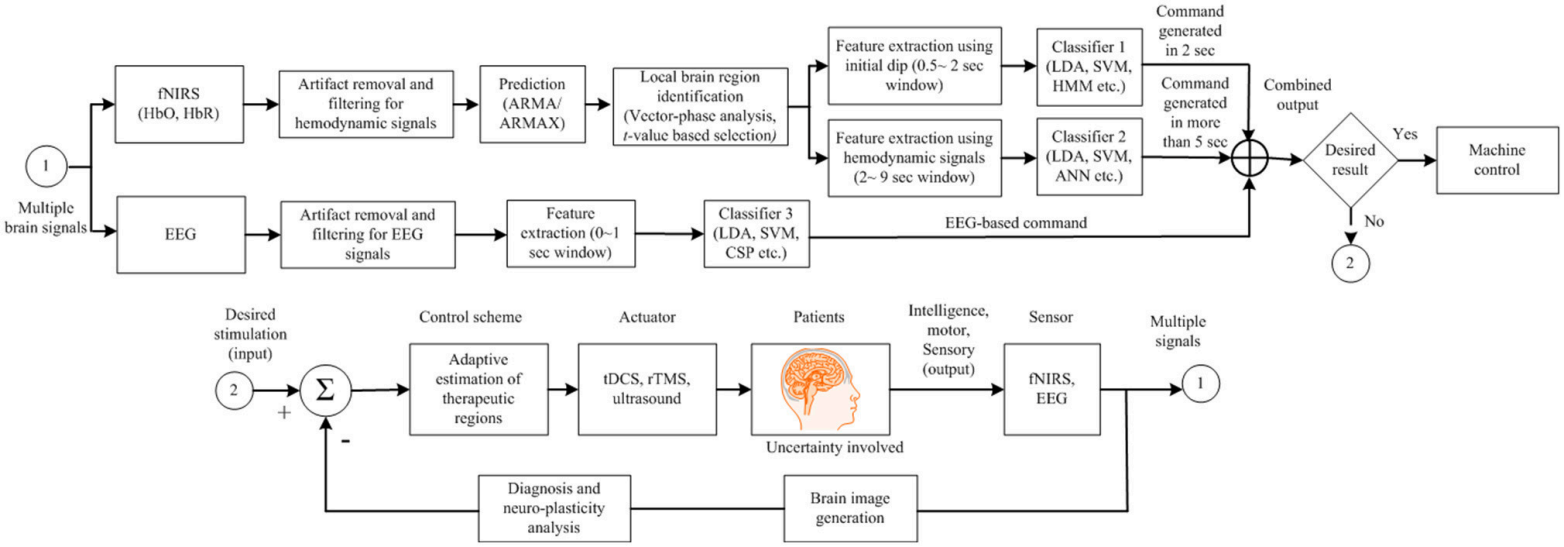
Proposed brain-computer interface (BCI) scheme to improve the BCI performance for device control for locked-in syndrome patients.

Brain therapy is also an important factor in the quest to improve brain state decoding. Many studies have shown that tDCS- and rTMS-based stimulation can significantly improve brain activity for purposes of BCI. In stroke patients, it is essential that the brain be monitored for signs of improvement (Sood et al., 2016). In our opinion, along with the use of hybrid brain-signal acquisition modalities, there is a need for continuous brain stimulation to improve the brain recovery process. Figure 9 shows our proposed scheme. As indicated in Figure 9, brain therapy is essential when brain activity detection is not workable. The correct brain region needs to be targeted for therapy. Integration of neuronal and hemodynamic signals may prove to be a good method for localizing it.

In comparison to the conventional fNIRS-BCIs (Blokland et al., 2014; Chaudhary et al., 2017), the suggested BCI scheme has an advantage of being more effective for patients. Currently, a specific brain region is not targeted and most BCIs are implemented by averaging the channels. For a patient, it is important to know the specific brain region that can be used for BCI. As per the authors' opinion shown in Figure 9, a specific brain region needs to be identified first for using EEG/fNIRS for command generation. If the activity recorded for BCI is not significant, a neuroplasticity in that brain region can be pursued. If the unresponsive brain region is identified by an adaptive algorithm, rTMS/tDCS stimulation can be applied to improve the neuroplasticity. Then, the brain region that shows improvement after brain stimulation can be used.

We suggest that the most pressing current need for fNIRS and hybrid EEG-fNIRS is to take experimentation out of the lab and test the results on real patients. To achieve this objective, a significant improvement in hardware development is needed. A hybrid system for hemodynamic and neuronal signal detection and integration is required in a package that is comfortable for patients (less bulky). The hardware should be able to handle the motion artifacts generated by LIS patients. Moreover, it is important to detect deep brain activation. Therefore, research should focus on development of hardware that can reliably acquire deep brain activity in real-time. Furthermore, algorithms are needed that can integrate the neuronal and hemodynamic signals to generate a reliable brain image. Perhaps, a breakthrough in fNIRS-BCI can be achieved by using brain imaging features instead of conventional features (Hong et al., 2017).

## Conclusions

In this paper, we have reviewed recent work on functional near-infrared spectroscopy (fNIRS) and hybrid fNIRS-EEG studies for brain-computer interfaces (BCI). The focus was on finding the brain activity patterns, channel selection criteria, feature extraction schemes, and classification algorithms that are most suitable for locked-in patients.

We discussed brain activities that can cause a significant increase in the hemodynamic response. We noted that mental arithmetic is the most widely used activity for fNIRS-BCI. However, there is no specific activity that can be claimed to be most suitable for locked-in patients. Similarly, there exist different algorithms for brain area identification, but their true potential for patients is yet to be seen. Moreover, signal mean and signal peak are the most appropriate features for classification of hemodynamics, but only a limited literature show evidences of these features giving good results for patients. For hybridization, most commonly the mean fNIRS signal is combined with the power spectrum density from EEG to improve the system accuracy. Lastly, linear discriminant analysis is the most widely used classifier for fNIRS and hybrid fNIRS-BCI. However, vector phase analysis is a new method that has potential both for identification of brain regions and classification of hemodynamic responses. Further research on this algorithm is warranted.

## Author contributions

K-SH has conceived the idea, corrected the manuscript, and finalized the work. MK conducted the literature survey and wrote the first draft of the manuscript. MH participated in revising the manuscript. All the authors have approved the final manuscript.

### Conflict of interest statement

The authors declare that the research was conducted in the absence of any commercial or financial relationships that could be construed as a potential conflict of interest. The reviewer NN declared a past co-authorship with one of the authors K-SH to the handling Editor.
